# Feasibility of Home-Based Automated Assessment of Postural Instability and Lower Limb Impairments in Parkinson’s Disease

**DOI:** 10.3390/s19051129

**Published:** 2019-03-05

**Authors:** Claudia Ferraris, Roberto Nerino, Antonio Chimienti, Giuseppe Pettiti, Nicola Cau, Veronica Cimolin, Corrado Azzaro, Lorenzo Priano, Alessandro Mauro

**Affiliations:** 1 Institute of Electronics, Computer and Telecommunication Engineering, National Research Council, Corso Duca degli Abruzzi 24, 10129 Torino, Italy; roberto.nerino@ieiit.cnr.it (R.N.); antonio.chimienti@ieiit.cnr.it (A.C.); giuseppe.pettiti@ieiit.cnr.it (G.P.); 2 Department of Electronics, Information and Bioengineering, Politecnico di Milano, Piazza Leonardo da Vinci 32, 20133 Milano, Italy; veronica.cimolin@polimi.it; 3 Istituto Auxologico Italiano, IRCCS, Department of Neurology and NeuroRehabilitation, S. Giuseppe Hospital, 28824 Piancavallo, Oggebbio (Verbania), Italy; n.cau@auxologico.it (N.C.); c.azzaro@auxologico.it (C.A.); lorenzo.priano@unito.it (L.P.); alessandro.mauro@unito.it (A.M.); 4 Department of Neurosciences, University of Turin, Via Cherasco 15, 10100 Torino, Italy

**Keywords:** Parkinson’s disease, UPDRS tasks, movement disorders, posture, postural stability, center of mass, RGB-depth, automated assessment, machine learning, at-home monitoring, neurorehabilitation

## Abstract

A self-managed, home-based system for the automated assessment of a selected set of Parkinson’s disease motor symptoms is presented. The system makes use of an optical RGB-Depth device both to implement its gesture-based human computer interface and for the characterization and the evaluation of posture and motor tasks, which are specified according to the Unified Parkinson’s Disease Rating Scale (UPDRS). Posture, lower limb movements and postural instability are characterized by kinematic parameters of the patient movement. During an experimental campaign, the performances of patients affected by Parkinson’s disease were simultaneously scored by neurologists and analyzed by the system. The sets of parameters which best correlated with the UPDRS scores of subjects’ performances were then used to train supervised classifiers for the automated assessment of new instances of the tasks. Results on the system usability and the assessment accuracy, as compared to clinical evaluations, indicate that the system is feasible for an objective and automated assessment of Parkinson’s disease at home, and it could be the basis for the development of neuromonitoring and neurorehabilitation applications in a telemedicine framework.

## 1. Introduction

Among chronic neurodegenerative diseases, Parkinson’s disease (PD) is recognized as the second most common disorder after Alzheimer’s disease. It causes an important negative impact on the quality of life characterized by a progressive impairment in motor functions [1].

Neurologists employ clinical assessment scales, such as the Part III of the Unified Parkinson’s Disease Rating Scale (UPDRS) [2], as a common basis to assess the motor impairment severity and its progression over time. During the patient assessment, particular features of the movements (e.g., amplitude, speed, rhythm, hesitations) or of the posture (e.g., trunk flexion, one-side leaning and posture recover capabilities) are subjectively evaluated by neurologists on a discrete scale of five classes of increasing severity, with reliability limitations due to intra and inter-rater variability [3]. Aiming to improve the clinical management and the quality of life of individuals with PD, more objective and automated approaches to disease assessment, also suitable for at home use, have been proposed.

The majority of these approaches employ wearable technologies [4,5], specifically in lower limbs UPDRS tasks assessment [6]; fewer of them are based on optical tracking, smartphones and other technologies [7,8]. Wearable and optical based technologies exhibit complementary aspects: the first ones are more ubiquitous but they are also more invasive and require more management efforts; the second ones are suited for spot assessment in a localized environment, but they are non-invasive. In general, for the assessment, both approaches make use of the correlation existing between the severity of the impairment, as assessed by UPDRS, and the static and/or kinematic parameters characterizing the pose and the movements [9,10]. 

Recently many low-cost, optical, body and hand tracking systems [11,12,13,14] have been employed successfully in the health care context. Among these, the Microsoft Kinect^®^ v1 device has been used to monitor people with PD [15], in rehabilitation [16,17], in body sway and balance [18,19], in gait assessment [20] and gait anomalies detection [21], in identifying different subjects by kinematic signature [22], in hand tracking [23] and to prevent falls [24]. The Microsoft Kinect v2 is more robust and accurate as compared to Microsoft Kinect v1 [25], and it has been recognized a viable tool for tracking human movement in clinical applications [26], standing balance and postural stability [27], gait [28], body sway [29] and clinical motor functions [30]. In the specific context of neuro-degenerative diseases, it has been used for time up and go test [31], in assessing different types of PD patients [32], to classify gait disorders [33] and in neurological rehabilitation [34].

The work presented here is part of a more extensive project aimed to bring UPDRS compliant automated assessments at patients’ home. In a former paper [35], we presented our work on the upper limb UPDRS tasks. Instead, in this case, the assessment is based on a Microsoft Kinect v2 device and it is focused on the analysis of posture and lower limb tasks, as specified by UPDRS [2]. This approach guarantees both to compare results with the standard clinical assessment scales, accepted and used by neurologists, and to define explicitly posture and movements to be performed. Furthermore, we select those UPDRS motor items which are suitable to be self-managed by patients at home, considering that some motor tasks are not feasible in any home environments. For example, according to task specifications [2], Gait task (UPDRS task 3.10) requires a safe straight walking path of 4–10 m, which is not usually available at home, as well as the Postural Stability task (UPDRS task 3.12) that cannot be self-administered being based on a retropulsion test. 

Specifically, we examine the following UPDRS tasks (Section 3, items 3.8, 3.9 and 3.13): Leg Agility (LA), Arising from chair (AC) and Posture (Po). Concerning Postural Stability UPDRS task (PS_retrop_, item 3.12), the standard retropulsion test used for the assessment is not a good predictor of fall risk. Furthermore, the related step count parameter is a too rough estimator of the postural instability [36]. Nevertheless, postural stability assessment is important to prevent falls and injury risk in PD, especially in advanced stages [37]. Postural stability deficits in PD subjects can be highlighted by concurrent cognitive tasks or by secondary motor tasks during steady standing stance tasks [38]. Several studies have found that, during the quite stance, the continuous movement of the center of mass (CoM), named “postural sway“ or “body sway”, contributes to balance control [39,40,41]. Alterations of body sway can reveal balance dysfunctions in PD, long before their clinical assessment [42] and they can be used to differentiate between motor subtypes of PD [43]. Recently, low cost RGB-Depth devices as Microsoft Kinect v2, have been used to assess objectively balance dysfunctions [44,45,46]. Given the importance of postural stability in PD progression and risk of fall prediction, we analyze the postural stability by CoM movements (PS_COM_) during the posture task. Furthermore, we investigate also the potential correlation between this method and a standard clinical measure of postural stability PS_PIGD_ based on the Postural Instability and Gait Difficulty (PIGD) subscale score of UPDRS, defined as the sum of the scores assigned to the AC, Gait, PS_retrop_ and Po tasks of UPDRS [47]. To our knowledge, this is the first time, in the context of PD, that a set of UPDRS tasks (namely, LA, AC, Po) and the Postural Stability (PS_COM_) during quite stance are automatically assessed by the use of low-cost optical body tracking devices. 

## 2. System Hardware and Software 

**Figure 1:**
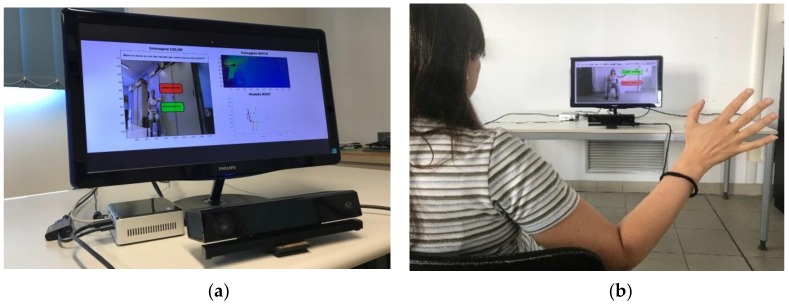
System for the lower limbs and postural tasks analysis: (**a**) RGB-Depth camera (Microsoft Kinect v2), NUC i7 Intel mini-PC and monitor (**b**) example of GUI with visual feedback.

The system hardware (Figure 1a) is built around a Microsoft Kinect v2 device (Microsoft Corporation, Redmond, WA, USA), which provides through its Software Development Kit (SDK) [12], RGB color and DEPTH streams at 30 frame/s, with resolution of 1920 × 1080 px and 512 × 424 px, respectively. The range of depth is from 0.5 m to 4.5 m. The device is connected, via an USB 3 port, to a NUC i7 Intel^®^ mini-PC running Windows^®^ 10 (64x) (Intel Corporation, Santa Clara, CA, USA) and equipped with a monitor to provide both a system management GUI and the visual feedback of the hand and body movements to the user (Figure 1b).

**Figure 2:**
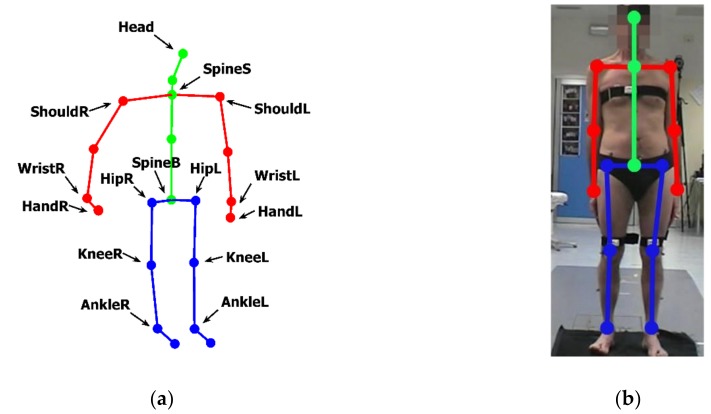
Positions of joints of the skeleton model from Microsoft Kinect SDK: (**a**) three-dimensional representation of joints and segments for body vertical axis (green), upper limbs (red), lower limbs (blue); (**b**) two-dimensional re-projection of the same joints and segments on the RGB image.

The system software is made by custom scripts, written in C++, which run on NUC and access the SDK APIs, providing every 1/30 s RGB images and 25 three-dimensional (3D) coordinates of the skeleton model used by the SDK (Figure 2). 

The data analysis and the supervised classifier training and testing phases are based on custom Matlab^®^ scripts (Mathworks Inc, Natick, MA, USA). The software implements different functionalities of the system: real-time interaction by a Human Computer Interface (HCI) based on hand joint tracking/processing and visual feedback; task movement analysis and characterization, by processing the 3D positions of specific task-dependent sets of skeleton joints; automated assessment of posture and lower limb tasks, through the implementation of trained supervised classifiers. Data of each acquisition session (consisting of video of each task performance, user inputs, trajectories of body movements and automated assessment scores) are encrypted and recorded to provide remote supervising facilities to authorized clinicians.

## 3. The Human Computer Interface 

**Figure 3:**
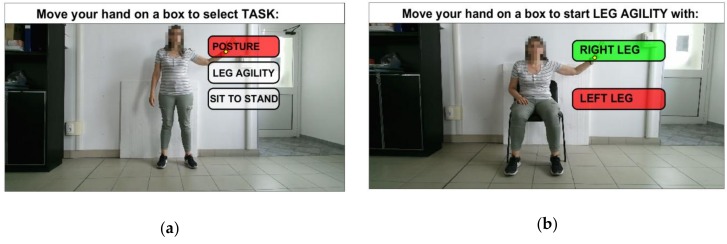
Gesture-based HCI: (**a**) GUI for the selection of lower limbs and postural tasks; (**b**) GUI for the selection of left/right leg before starting LA task.

The HCI provides a natural interface suitable for subjects with limited computer skills and with motor impairments. It is implemented through a Graphical User Interface (GUI) and an interactive menu based on choice icons (Figure 3). During the interaction with the system, the user is guided by video and textual support. The 3D position of HandR joint, output by SDK, is tracked and re-projected onto the GUI screen, and the user selection is confirmed by considering the hand closure information provided by the SDK (Figure 1b, Figure 2a and Figure 3). At any time during an assessment session, the user can stop it and quit, for example when tired, to avoid the onset of stress and/or anxiety. 

## 4. Automated Assessment of UPDRS Tasks

### 4.1. Participant Recruitment 

Two cohorts of subjects, consisting of fourteen PD patients and twelve Healthy Controls (HC) respectively were recruited. The PD patients were assessed for the LA, AC, Gait, PS_retrop_ and Po tasks (UPDRS tasks 3.8, 3.9, 3.10, 3.12 and 3.13, respectively) by two neurologists (N1, N2) expert in movement disorders. The postural stability score (PS_PIGD_) was assessed by the PIGD subscale score obtained from the AC, Gait, PS_retrop_ and Po UPDRS tasks [47]. Motor impairment is sensitive to the time passed after the last drug intake; therefore, the OFF state (practically defined as that after 12 h without medication) was chosen as the reference for disease severity scoring. PD patients were excluded if they had previous neurosurgical procedures, tremor severity > 1 or cognitive impairment (Mini–Mental State Examination Score < 27/30). PD patients met the following criteria: Hoehn and Yahr average score 2.1 (min 1, max 3); age range 53–80 years (mean 69, std. dev. 7.5). disease duration range 3–10 years (mean 5.8, std. dev. 2.5), gender 8 men and 6 women. The HC subjects performed the same tasks, in the same environmental conditions and with the same system setup of PD patients. The HC cohort was selected trying to approximately match the PD cohort in age and gender, excluding subjects affected by neurological, motor and cognitive disorders. 

Informed consent was obtained in accordance with the Declaration of Helsinki (2008). The study’s protocol was approved by the Ethics Committee of the Istituto Auxologico Italiano (Protocol n. 2011_09_27_05).

### 4.2. Experimental Setup

**Figure 4:**
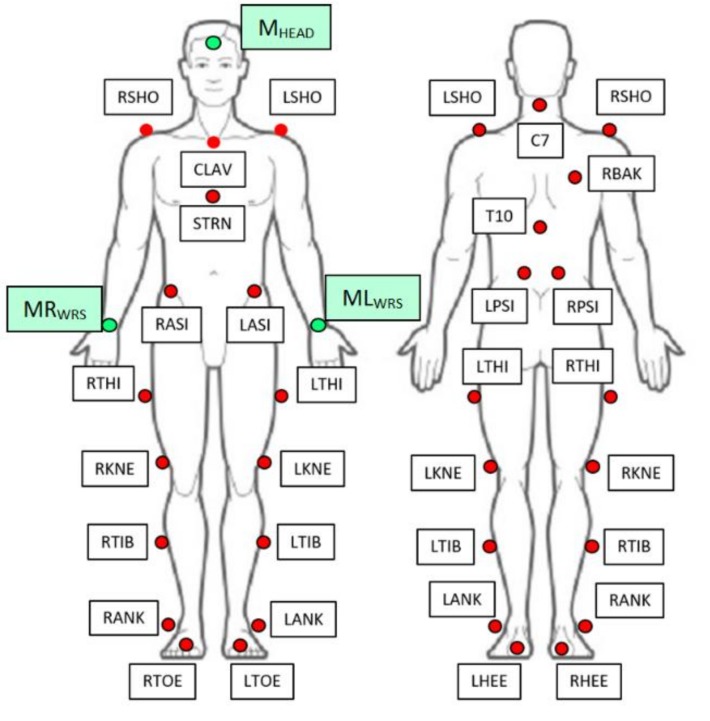
Details of the marker set placement positions.

An experimental setup has been built both to assess the accuracy of the system and to acquire relevant clinical and kinematic data useful for the automated assessment of the UPDRS tasks. The kinematic parameters evaluated by the system were compared with those evaluated by an optoelectronic system, considered as gold reference (BTS SMART DX400^©^, eight TVC, 100–300 fps, BTS Bioengineering, Milan, Italy) [48]. For this experiment, reflective markers were attached to the body of the PD and HC subjects to evaluate kinematics of lower limbs, thorax, spine and head (Figure 4). The biomechanical measurements of the lower body were modelled according to the Helen Hayes Marker set [49,50] and those of the upper body were modelled according to the Plug In Gait model (Vicon^®^ Motion Systems, Oxford, UK) [51], focusing the attention only on thorax, shoulders and spine. Three additional markers were put on the forehead (M_HEAD_), on the right (MR_WRS_) and the left wrist (ML_WRS_) respectively, this to allow for the assessment of the head posture, CoM estimation and data synchronization between our system and the optoelectronic system. The body markers relevant for the accuracy assessment and their reference positions are presented in Table 1. 

### 4.3. Data Acquisition Procedure

During the experimental campaign, the HC and PD subjects were equipped with the set of reflective markers shown in Figure 4, and were instructed to perform the UPDRS tasks. Their performances were supervised and assessed by two neurologists and simultaneously acquired by the two systems. The neurologist’ scores, the kinematic parameters and the videos of each task performance were recorded for the subsequent analysis.

The PD and HC subjects performed all the tests facing our system, that is, with the depth axis of the Kinect device perpendicular to the subject frontal plane. The proper position of the subject was verified both by the neurologists and by the system software, which checks the availability of the whole Kinect skeleton and its correct positioning. The device was placed 1.2 m height and at a distance of about 2 m from the subjects. No other object apart the chair, and limited to the AC and LA tasks, was allowed in the working volume. At the beginning of each test, the subject elevated the right arm three times, this to allow the synchronization between the two systems. The synchronization was performed by time-shifting the signals of the arm elevation angles measured by the systems such that their cross-correlation was maximized. The PD and HC subjects were told to perform the LA, AC and Po tasks as indicated by the UPDRS guidelines. Furthermore, the PD subjects performed also the Gait and the PS_retrop_ tasks, in order to evaluate the PIGD sub-score. For the LA task both legs were assessed independently. Two acquisition sessions were planned separated by an interval of thirty minutes to allow subjects to rest. 

In the first session a total of five tasks were assessed by the neurologists for the fourteen PD subjects, and a total of three tasks were supervised for the twelve HC subjects. In the second session the same tasks of the first one were repeated in random order. The body sway of the CoM movements was measured during the Po task: in the first phase (indicated as Phase1) each subject was told to stand up straight for ten seconds. Then, during the second phase (indicated as Phase2) each subject was told to try to improve and maintain a more straight posture for other ten seconds: this can be considered a sort of secondary motor task, that potentially can highlight differences between PD and HC subjects [38].

### 4.4. Movement Characterization by Kinematic Parameters

**Figure 5:**
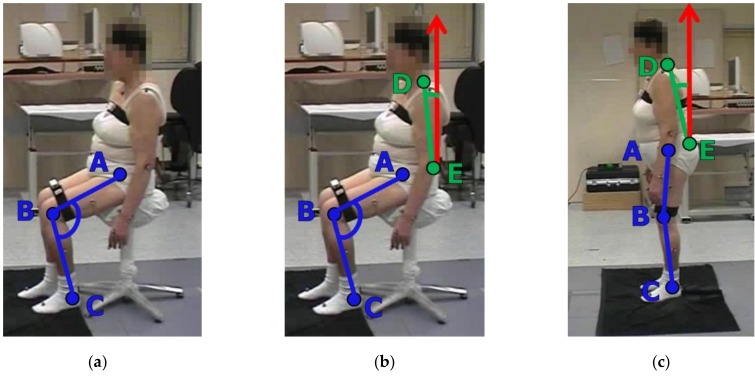
Segments involved in the estimation of the angular measures during lower limbs and postural tasks: (**a**) LA task (**b**) AC task (**c**) Po task. Note that the depth axis of the Kinect device is perpendicular to the subject frontal plane in all the tests, see Section 4.3).

The analysis and the related characterization of the considered UPDRS tasks make use of kinematic parameters which are mainly estimated from angles between pairs of body segments, involving femur, knee, tibia, spine and head. The body segments are defined by their distal and proximal points, which in our system are assumed to correspond to the joints of the skeleton model of Figure 2. The centroid of each segment is calculated as the midpoint between the proximal and distal extremities. The postural stability is assessed by the body CoM, which is estimated by the weighted average of the body segment centroids. In particular, the kinematic characterization of the LA, AC and Po tasks is based on the evaluation of the angles ANG_KNEE_ and ANG_TRUNK_. Only for the Po task two further angles are considered: the forward ANG_FORHEAD_ and lateral ANG_LATHEAD_ bending angles of the head respect to spine direction. Specifically, with reference to Figure 5 for the proximal and distal 3D points relevant for the analysis, and to Figure 2a for the 3D skeleton joints involved, we considered:

- For the LA task (Figure 5a): the knee angle ANG_KNEE_ between the A-B and B-C segments, with A = HipR, B = KneeR and C = AnkleR for the right limb, and A = HipL, B = KneeL and C = AnkleL, for the left limb;
- For the AC task (Figure 5b): the knee angle ANG_KNEE_ defined above; the trunk angle ANG_TRUNK_ between the D-E segment and the vertical direction $\hat{n}$ (i.e., the red arrow), with D = SpineS and E = SpineB;
- For the Po task (Figure 5c): the knee angle ANG_KNEE_ and the trunk angle ANG_TRUNK_ defined above. The bending of the head respect to spine is evaluated by the angles ANG_FORHEAD_ and ANG_LATHEAD_, projections of the angle between the SpineS-SpineB (D-E) segment and the SpineS-Head segment on the sagittal and frontal body planes, respectively. The lateral body plane is approximately identified by the plane containing the segments ShouldR-ShouldL and SpineB-SpineS, while the sagittal body plane is perpendicular and contains the SpineB-SpineS segment. Note that while ANG_TRUNK_ has components in the sagittal and lateral body planes, ANG_FORHEAD_ and ANG_LATHEAD_ have components only in the sagittal and in the lateral planes, respectively.

The CoM is estimated during the Phase1 and the Phase2 of the Po task, both to evaluate the postural instability and to evidence the effects of the secondary tasks. A subject specific quasi-static center of mass **C**_b_ is evaluated by applying the kinematic method described in [52,53]. As indicated in Equation 1, **C**_b_ is obtained by the weighted average of the body segment centroids (**C**_i_), evaluated from the skeleton model, where the weights w_i_ are provided by standard body segment densities obtained from anthropometric data [54]:

(1) $$C_{b}=\;\frac{1}{N}\;\overset{N}{\underset{i=1}{\sum }}C_{i}*\;w_{i}$$

The centroids **C**_i_ of the following segments made by pairs of skeleton joints are considered (Figure 2a): Head-SpineS, ShouldR-WristR, ShouldL-WristL, SpineS-SpineB, HipR-AnkleR, and HipL-AnkleL. Please note that **C**_b_ is a 3D point; but here only the transverse (or horizontal) plane components are evaluated for the analysis of the body sway. Concerning the evaluation of the kinematic parameters, the skeleton joints provided every 1/30 s by the Kinect SDK allow the estimation of the relevant parameters at the same rate. In particular, the angles ANG_KNEE,_ ANG_TRUNK_, ANG_FORHEAD_ and ANG_LATHEAD_ were evaluated from the inner products of the pairs of unity vectors representing to the body segments involved. The vertical direction $\hat{n}$, used to evaluated ANG_TRUNK_, was estimated by the normal to the floor plane. The 3D orientation of the plane was obtained by segmentation of the Kinect depth map using a RANSAC approach [55], with the upside direction of the Kinect skeleton and the feet location as priors. The angle signals were resampled both to remove the typical jitter of the Kinect sampling frequency, and to fit the sampling frequency of the optoelectronic system (100 Hz). The signals are filtered to reduce noise by a second order low-pass Butterworth filter with a cut-off frequency of 10Hz. Most of the significant kinematic parameters presented in the Results and used as input to the classifiers were obtained by standard signal processing algorithms applied to the sampled signals of the ANG_KNEE_, ANG_TRUNK_, ANG_FORHEAD_ and ANG_LATHEAD_ angles. The velocity parameters were evaluated as the derivatives of the spline approximations to the angle signals obtained through Matlab functions (unmkpp, mkpp and ppval). Specifically:

- For the LA task, the ANG_KNEE_ signal is segmented in a sequence of flexion/extension movements (cycles) by finding all the minimum-maximum-minimum sequences in the amplitude of the angle signal. The peak to peak amplitude, the speed and the duration of every flexion/extension movement of the leg is evaluated. Specifically, MKAm is the mean of the peak to peak amplitude maxima and MKAv is its standard deviation; TDm is the mean of the cycle durations and TDv is its standard deviation; SPm is the mean of the speed maxima. Finally PM is the number of “poor movements”, defined as the cycles whose amplitude and duration are both less than 25% of the MKAm and the TDm values. This last parameter tries to catch hesitations and very small amplitude cycles in a sequence of almost relevant cycles.
- For the AC task, the ANG_TRUNK_ signal is segmented in a sequence of forward/ backward bending movements (cycles) by finding all the minimum-maximum-minimum sequences in the amplitude of the angle signal. Usually, only one peak is present, but hesitation during the movement or some instability event can generate other peaks in the signal. These peaks are clinically relevant and, consequently, have been considered in the assessment through the parameter NPeaks. MBA is the maximum angular peak and TD is the duration of the main bending cycle containing MBA, while SPm is the mean speed during the AC movement.
- For the Po task, during the quasi-static Phase1 are evaluated the bending angle FTB of the spine respect to the vertical (mean of ANG_TRUNK_), the forward bending angle FHB (ANG_FORHEAD_) and the lateral bending angle LHB (ANG_LATHEAD_) of the head. During the Phase2, the variations of these angles (FTB_Δ_, FHB_Δ_, LHB_Δ_) respect to Phase1 are evaluated.
- For the PS_COM_ task, the sway is defined as the CoM component in the transversal plane (perpendicular to both the lateral and the sagittal body planes). In this plane, the Antero-Posterior (AP) and Medio-Lateral (ML) axis are defined as the intersection of sagittal and lateral planes with the transversal plane, respectively. The AP components of the range, total path length and velocity of the sway (APr, APt and APv, respectively), and the ML components of the range, total path length and velocity of the sway (MLr, MLt and MLv, respectively) are evaluated. Furthermore, the sway area SwayArea (convex hull of the sway path) is also evaluated.

The Pearson’s correlation between the measures of the kinematic parameters provided by our system and those measured by the optoelectronic system was used to assess the body tracking accuracy. Because all the kinematic parameters for LA, AC, Po and PS_COM_ were obtained from the ANG_KNEE_, ANG_TRUNK_, ANG_FORHEAD_ and ANG_LATHEAD_ angles and from the CoM components in the transversal plane, only these last “essential” parameters were considered for the accuracy assessment.

The correspondences between optoelectronic markers (Figure 4 and Table 1) and Kinect joints (Figure 2a) we adopted for the comparison are shown in Table 2. The optoelectronic angular parameters corresponding to the essential ones were obtained by the marker correspondences of Table 2 and by the same procedure we used for the Kinect joints in Section 4.4. The CoM measured by the optoelectronic system was evaluated according to [51].

### 4.5. Discriminant Parameter Selection

The choice of the best parameters used to train the classifiers was performed by selecting the sets of kinematic parameters which best correlate with the UPDRS scores of subjects’ performances. 

The initial sets of parameters considered to characterize every single task consisted of more than ten parameters per set: they were chosen to be closely related to those features that are implicitly considered by neurologists to assess the motor performance. These initial sets could potentially include irrelevant and redundant parameters, which could hide the effects of the clinically relevant ones, reducing the predictive power of the classifiers used for the automated assessments. To avoid this, a feature selection (FS) procedure [56] is performed by the Elastic Net (EN) algorithm [57]. EN is a hybrid of Ridge regression and LASSO regularization. EN encourages a grouping effect on correlated parameters, and tends to be more conservative respect to LASSO or Ridge regression in removing correlated parameters, a process which can select incorrect data model. This capability is important when dealing with those features which are similar and tend to be moderately correlated. The EN implementation is based on Matlab scripts (lasso Matlab function). To avoid biasing the results by the different scaling, the PD parameters *p*_i PD_ have been normalized (Equation (2)) by the corresponding average values of the HC parameters *p*_i HC_. Then the normalized parameters range from the value 1 (*p*_i HC_ ) to a maximum (*p*_i PD Norm MAX_ > 1), or to a minimum (0 < *p*_i PD Norm MIN_ < 1), depending if the value of the specific parameter increases or decreases when the severity of the impairment increases. The parameter Number of Poor movements (PM), whose minimum value is 0, was not normalized:

*p*_i PD Norm_ = *p*_i PD_/*p*_i HC_, (2)

### 4.6. Statistical Analysis 

Descriptive statistical analysis of the collected data by Mann-Whitney U and χ^2^ tests did not show significant differences among PD and HC (age, gender, cognitive status), then the data were safely pooled into two groups (PD and HC) for the following analyses.

We note that, as common in feature selection algorithms, the previous selection of parameters based on the EN algorithm assumes the UPDRS scores are ratio data type, while they actually are ordinal data. Then, to confirm the relevance of these parameters in the context of the ordinal nature of the scores and to deal with their non-normal distributions, Spearman non-parametric rank correlation at a significant level *p* < 0.05 was applied. Only those parameters showing a Spearman’s correlation coefficient ρ greater than 0.3 (as absolute value) with respect to the UPDRS scores assigned to the LA, AC and Po task performances were considered for the final sets. For the PS_COM_ task, the CoM parameters were correlated with the PIGD subscale scores (PS_PIGD_).

Furthermore, as a support to the effectiveness of the selected parameters in the automated assessment, the statistical significance of each parameter in discriminating PD and HC was considered and verified by the Mann-Whitney U test with *p* < 0.05. All statistical analyses were performed using Matlab. For the correct application of the test, only the data of the second acquisition session were considered.

### 4.7. Supervised Classifier Training 

Three different types of supervised classifiers have been considered for the automatic assessment of the LA, AC, Po and PS_COM_ tasks: k-Nearest Neighbours (kNN), Multinomial Logistic Regression (MLR) and Support Vector Machine (SVM) with polynomial kernel [58]. Two types of classification problems were considered: first, a binary classification problem, where the subjects are classified into the HC and the PD classes; second, a multiclass classification problem, where the subjects are classified into the three PD classes of increasing severity. The design of this second experiment was suggested by the distributions of the severity scores of the PD patients recruited for the study, which were essentially distributed among slight, mild and moderate UPDRS severity scores [2], corresponding to the UPDRS1, UPDRS2 and UPDRS3 classes, respectively. Furthermore, the severity scores distributions were adequately balanced among the classes for all the tasks (Table 3). The classifiers were trained for each task (LA, AC, Po, and PS_COM_) by using as input the sets of “selected kinematic parameter vector—UPDRS score” pairs obtained from the reference dataset of performances. In particular, the PS_COM_ classifiers was trained using the PIGD subscale scores (PS_PIGD_) as UPDRS score.

The input data have been normalized both to make more stable the training procedure and to simplify the behavior of the parameters in the parameter space. Specifically, the parameters *p*_i PD Norm_ whose values increases with the worsening of the performance from 1 to their maximum (*p*_i PD Norm MAX_ > 1), are scaled in the range 0 to 1, while those whose values decrease with the worsening of the performance from 1 to the their minimum (0 < *p*_i PD Norm MIN_ < 1) are first reversed and then scaled in the range 0 to 1. The score values are scaled in the range 0 to 1 as well. 

The kNN classifiers were employed as baseline and implemented and tested by Matlab scripts (fitcknn function). The classifiers were tested with parameter k = 1,3,5,7 using the Euclidean distance metric. The tie breaking algorithm adopted was to decrease k by 1 until the tie is broken. 

The MLR classifiers were implemented and tested by Matlab scripts (fitmnr function for ordinal data with probit link function).

The SVM classifiers were implemented and tested by Matlab scripts with the support of the LibSVM library package [59]. The kernel function of SVM classifier is polynomial with parameters: γ (gamma), *r* (bias) and *d* (polynomial degree) and *C* (cost). Every SVM multiclass classifier uses the one-versus-one coding design with majority voting scheme and is made by three binary SVM models, all with the same parameters [60]. A grid-search and cross-validation method were used to find the optimal values of the SVM parameter *C*, γ, *r* and *d* for the three binary classifiers.

### 4.8. System Reliability and Accuracy Evaluation 

A commonly accepted measure of reliability in the context of clinical assessments is the Intra Class Correlation coefficient (ICC). Accordingly, the reliability of the system assessments respect to the neurologist ones was evaluated by the Intra Class Correlation coefficient ICC_N12-SY_ (two-way random effects model with an absolute agreement) [3]. The inter-rater agreement ICC_N12_ between the two neurologists was evaluated and compared as a baseline with the inter-rater agreement ICCN_12-SY_ among neurologists and system, considering the system as a third “virtual” neurologist. 

In the evaluation of ICC_N12_, the scores of the neurologists for the LA, AC, Po tasks and for the subscale PS_PIGD_ were considered, while for ICCN_12-SY_ both the neurologist scores and the corresponding system scores were used. Concerning the reliability of the remote video-based assessments, motor examination of video recorded UPDRS tasks has already been demonstrated to be a sufficiently accurate alternative to in field ones [61]. In machine learning context, it is more common to assess the reliability of classifiers by their accuracy. Then, we evaluated also this measure of system performance considering the mean accuracies of each classifier, both in discriminating between PD from HC subjects (binary classification problem) and in classifying PD subjects into different severity classes (multi-classes classification problem) [62].

## 5. Results

### 5.1. Clinical Assessment Results

After collecting the clinical assessments at the end of the experiment, none of the performances of the PD cohort were scored with normal (score 0) or severe impairment (score 4) for all the UPDRS tasks considered. The distributions of the severity scores assigned to the PD patients among slight, mild and moderate responses were relatively balanced for all the tasks (Table 3). 

### 5.2. Accuracy of the Kinematic Parameter Evaluation

The measurement accuracy of Microsoft Kinect v2 in clinical estimation of motor functions [27,28,30] and body CoM [18,19,25,27,29] has been previously assessed: this was confirmed also by our experiment. The comparison of the parameter measurement respect to the gold reference system cannot be performed directly because the Kinect skeleton model and the optoelectronic marker set have different body landmark positions. Furthermore, for every parameter, we want to estimate an average accuracy based on all the trials per task acquired. 

**Figure 6:**
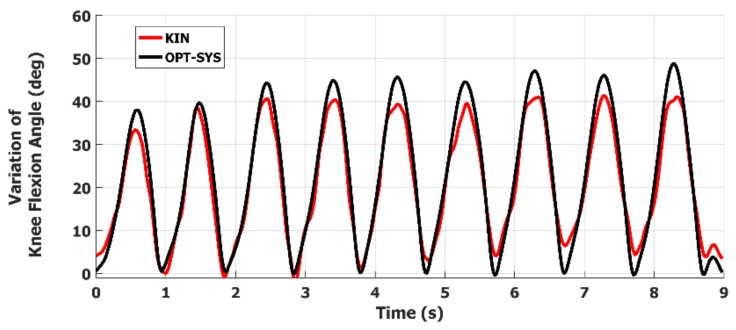
Example of the ANG_KNEE_ variations during the LA task performance of a PD subject: the last movement at 8.9 s is characterized by significant reduction in both amplitude and duration.

**Figure 7:**
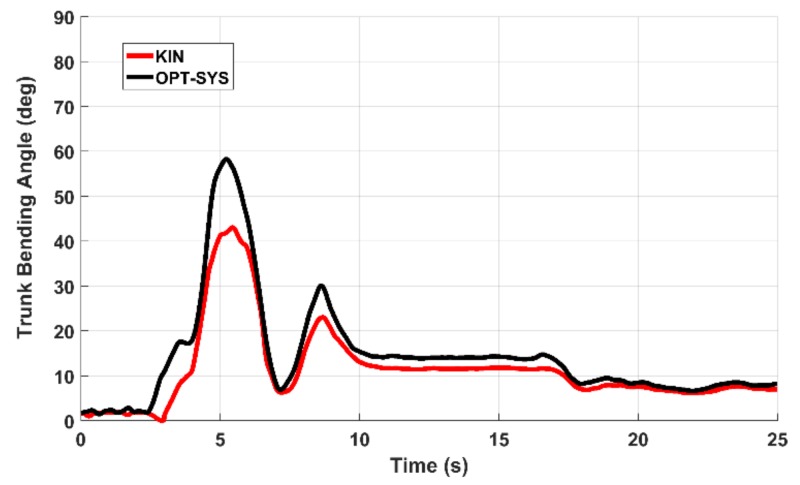
Example of the ANG_TRUNK_ variations during the AC task performance: the secondary peak at 8.5 s indicates the presence of an instability event in the final standing stance.

Then, for every essential parameter *i*, characterizing one or more tasks, the parameter samples of each associated task trials were joined together into a single parameter sample sequence (PSS_i_). The PSS_i_ sequences measured by the two systems were then compared by evaluating the Pearson correlation coefficient *r_i_*. In Table 4, the *r_i_* coefficients indicate a significant correlation that ranges from good to strong for all the examined parameters. In Figure 6 and Figure 7 are shown two examples of the ANG_KNEE_ and the ANG_TRUNK_ variations for a LA and an AC task trial. In Figure 6, it is interesting to point out that the last movement is characterized by a significant reduction in amplitude and duration. In Figure 7, the presence of a secondary peak indicates that the PD subject had an instability event at the corresponding time. These anomalies are hardly identified by neurologists.

### 5.3. Discriminant Kinematic Parameter Selection and Validation

**Figure 8:**
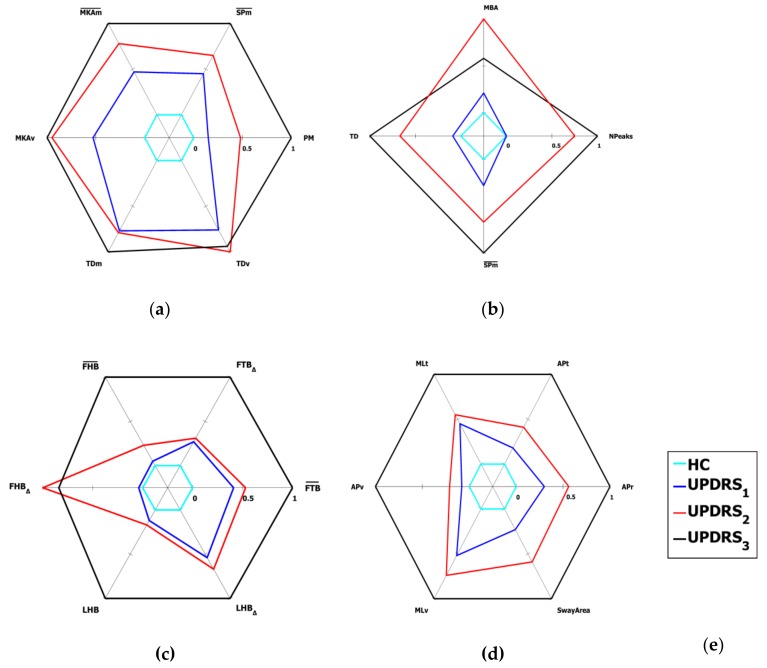
Radar graphs of the mean values of the normalized kinematic parameters of HC and UPDRS severity classes for the lower limbs and postural tasks: (**a**) Leg Agility (LA); (**b**) Arising from Chair (AC); (**c**) Posture (Po); **(d)** Postural Instability (PS_COM_); (**e**) Legend for the radar plots. See Section 5.3 for further details of the graph representation.

The Spearman correlation values between selected parameters and UPDRS scores, and the Mann-Whitney U test values concerning their significance in discriminating PD and HC subjects are shown in Table 5, Table 6, Table 7 and Table 8 for the LA, AC, Po and PS_COM_ tasks, respectively. We remark that, for PS_COM_ task, the Spearman correlation was evaluated respect to the PS_PIGD_ subscale scores. For the correct application of the U test, only the data of the second acquisition session were considered. The results in the tables show that all the selected parameters correlate with UDRS score ($\left|\rho >0.3\right|$, *p* < 0.05). Furthermore, they are all significant for Mann-Whitney test (*p* < 0.05), even though at different significance levels. The mean values of the selected parameters respect to the UPDRS severity classes are shown in the radar graphs of Figure 8 for all the tasks. 

The parameters have been represented such that an increasing values indicate a worsening of the performance, highlighted by a corresponding expansion of the related graph. For this reason, the parameters of Table 5, Table 6, Table 7 and Table 8 are represented in Figure 8 directly (with the original parameter name) or inversely (with an overscore on the original parameter name), depending if the parameter value increases or decreases when the severity of the impairment increases.

Furthermore, with reference to Section 4.5, the parameters are scaled in such a way that the parameter values corresponding to the best performance (*p*_i PD Norm_ = *p*_i HC_ ) are represented on the innermost circle (i.e., value = 0) and those corresponding to the worst one (*p*_i PD Norm MAX_, or 1/*p*_i PD Norm MIN_, depending on the parameter) are represented on the outermost circle (i.e., value = 1). 

Finally, it should be noted that almost all the parameters are able to discriminate the different UPDRS classes for the LA, AC, Po and PS_COM_ tasks, pointing out the increasing severity of motor impairment by the corresponding increasing of their values. The graphs are encapsulated and do not overlap, which means that a monotonic increase of the parameter value corresponds to an increase (and so a worsening) of the UPDRS score.

**Figure 9:**
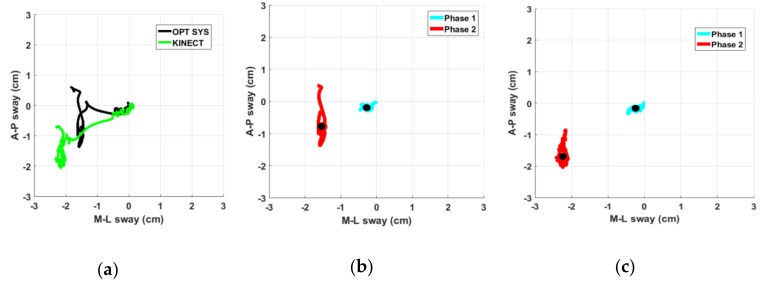
(**a**) Example of CoM trajectory of a PD subject represented in the Antero-Posterior (AP) and Medio-Lateral (ML) components during the Po task, as measured by our system (green line) and by optoelectronic system (black line); (**b**) Details of the trajectories during the first (cyan line) and second phase (red line) of PS_COM_ task with the respective centroids (black dots) as measured by optoelectronic system; and (**c**) as measured at the same time by our system.

The Pearson correlation analysis of the CoM movements, as measured by our system and by the optoelectronic system, shows that they are correlated both in the Antero-Posterior (AP) and in Medio-Lateral (ML) components (Table 4). These values confirm the feasibility of Kinect in the accurate estimation of center of mass movements. In Figure 9a, an example of CoM trajectories as measured at the same time by the two systems is shown; the trajectory of center of mass resembles the gold reference one, even if a scale factor is present. Figure 9b shows an example of the two phases of PS_COM_ task: in particular, the CoM trajectory measured by the optoelectronic system while a PD subject is performing the Phase1 (solid cyan line) and the Phase2 (solid red line) respectively. In Figure 9c, the same movement as measured by our system is shown. 

In both figures, the secondary motor task (during which the PD subject is trying to improve and then maintain a straighter posture) clearly increases the body sway along the AP direction, supporting the hypothesis of a performance degradation for PD subjects respect to HC in this context. The shapes of trajectories are quite similar: this confirms the feasibility of our system in acquiring the body CoM in agreement with the gold standard. Again, there is a mild scaling and an offset between the centroids of the trajectories measured by the two systems: this is probably due to the different landmark positions of the body skeleton models considered and to the different algorithms used to estimate the CoM position. Nevertheless, we remark that the CoM parameters we chose are independent from these biases. Furthermore, they convey useful information which well correlates with clinical evaluations, discriminating between PD from HC subjects, as indicated in Table 9. This is evident for almost all the PD subjects, on AP and/or ML directions; on the contrary, this is negligible for HC subjects, as confirmed by the values in the second and third column of Table 9. Furthermore, the differences of the CoM parameters (Phase2 respect to Phase1) between PD and HC subjects are significant at level p < 0.05, both for the U test and for the T test (column 5 and 6, Table 9). 

### 5.4. Reliability of the Assessments of the System and the Neurologists 

The values for the inter-rater agreement between the neurologists N1 and N2 (ICC_N12_) and among the neurologists and the system (ICC_N12-SY_) are shown in Table 10 (ρ values and 95% confidence intervals). According to [3], the ICC values less than 0.5, between 0.5 and 0.75, between 0.75 and 0.9, and greater than 0.90 are indicative of poor, moderate, good, and excellent reliability, respectively. In the evaluation of the ICC_N12-SY_ for PS_PIGD_, we use the system evaluated PS_COM_, stressing both the interpretation of PS_COM_ as a posture stability score and its good correlation with PS_PIGD_. 

The ICCN_12_ values, as reported in Table 10, indicate a generally good agreement between the two neurologists, with differences per task compatible with literature results. The results for ICCN_12-SY_ show the system does not degrade significantly the inter-rater agreement between the neurologists, except for PS_PIGD_. This could be due to the limited number of subjects examined, or to the use of CoM parameters not completely superimposable to PIGD subscale assessments. In fact, CoM parameters are evaluated only in PS_COM_ task, e.g., during postural adjustments from the quiet stance and not in other more challenging dynamic domains of postural stability characterization [37].

### 5.5. Accuracies of the Supervised Classifiers 

The classification accuracies of the LA, AC, Po and PS_COM_ tasks are shown in Table 11. They are obtained applying the leave-one-out and 10-fold cross validation method for the MLR, SVM and kNN classifiers. Accuracies refer to two different classification goals per task: discriminating PD from HC (two-classes classifier, binary problem) and classifying PD subjects into three UPDRS severity classes (three-classes classifier, multiclass problem).

The kNN with k = 3 and SVM with polynomial degree d = 2 gave the best performance using the leave-one-out cross validation, then these values were chosen for the system classifiers. In general, the accuracies of SVM classifiers are better than the kNN and MLR ones. Furthermore, the results of binary classification problem, in classifying HC and PD subjects, are quite better than the multiclass classification ones. This behavior was not unexpected because, in general, the classifiers perform worse on the same training data when the number of classification labels (i.e., classes) increases. In reporting the multiclass classification accuracy is more appropriate to indicate the per-class accuracy (fourth column for leave-one-out and sixth column for 10-fold of the Table 11), where the classification accuracies are averaged over the classes [62]. The absolute classification error (e_c_) was defined as the difference between the UPDRS class C, assigned by the neurologists, and the estimated class C’ assigned by the system to each motor performance *i* (e_c_ = |C*i* – C’*i*|). The e_c_ value for the kNN and MLR classifiers is sometimes larger than 1 UPDRS class, even when their average accuracies are better than that of the SVM classifiers. On the contrary, the e_c_ value for the best SVM classifiers was never greater than 1 UPDRS class for all the tasks; this means that the automatic assessments are always close to the neurologist’ ones. This is also an important feature for the system reliability respect to an average greater agreement but with large spot disagreements.

In addition, the results in Table 11 show that the two-classes accuracy is higher for LA and PS_COM_, while is slightly lower for the other two tasks. This is in agreement with the Figure 8, in which the AC and Po graphs show more overlapping between UPDRS classes as compared to the LA and PS_COM_ ones. The partial incoherence of some parameters in separating the different classes has probably an impact on the classifier performance. The behavior of the two-classes classification accuracy is not repeated in case of the three-classes classification, for which the worst performance is obtained for PS_COM_ task. This could be due again to CoM parameters not directly comparable to PIGD subscale assessments. Looking at the error distribution, we obtain a big contribution from UPDRS 3 class (i.e., most impaired PD subjects). The limited number of observations assessed as UPDRS 3 suggests that some significant parameters, which should have been considered, are probably missed, and could be included among the selected features only by increasing the number of UPDRS 3 observations in the training set.

## 6. Discussion

The availability of low-cost home-based solutions for the reliable and automated assessment of motor symptoms in Parkinson’s disease is highly desirable since it could provide several advantages, among which: reduction of costs and patient discomfort; better and prompt supervising and adjustment of the therapy; healthcare analytics for patient care improvements. Surely, among the features that these solutions should exhibit, particularly important are: a non-invasive approach to the assessment; a user-friendly interaction suitable to motor impaired users; an objective, continuous and automated evaluation of patient status, strongly correlated with the standard clinical assessments; an improvement of the reliability respect to the typical intra and inter-rater variability of the clinical evaluations. 

In this paper, a self-managed system for the automated assessment of Parkinson’s disease which tries to implement many of the aforementioned features is presented. The developed system is focused on posture instability and motor impairments of lower limbs and it is one of the elements of a larger project aimed to bring an overall automated assessment of UPDRS tasks at home [35].

As a first step, we addressed both the non-invasiveness and user-friendly interaction by a low-cost system based on an RGB-D optical device, then providing a gesture based human computer interface for the self-management of the assessment procedures. The usability of the interface was tested and verified by PD users during a campaign of data acquisition sessions. Then, the accuracy of the kinematic measures, as obtained by the system, was validated successfully by comparison with a gold standard equipment (i.e., an optoelectronic system). This was a necessary preliminary requirement, since an objective evaluation of the patient status is based on the strong correlation existing between motor impairments and kinematic parameters extracted from patient’s movements.

To reliably refer the system assessments to the clinical ones, the analysis of possible movements was constrained to those specified by the UPDRS tasks. An experimental protocol was designed in which PD patients and healthy controls were assessed at the same time both by neurologists and by the system during the execution of the specific standard tasks defined by UPDRS. A feature selection procedure yielded to sets of optimal parameters, both correlated to UPDRS clinical scores and statistically significant in discriminating PD subjects from healthy controls. As shown in Figure 8, not all these parameters have the same discriminant power to separate subjects among the different PD severity classes; this is true especially for the AC and Po tasks. This is probably due to the limited number of PD subjects examined: consequently, further experiments could improve the current results. 

Following related works based on wearable systems [40,41], the postural stability of PD subjects was characterized by CoM movements. We analyzed the CoM trajectories during the two phases of the Po task (named PS_COM_), assuming the Phase2 as a mild secondary motor task [38]. As in [40,41], large differences in CoM trajectories of PD respect to HC were found. Differently to [41], a good correlation between PS_COM_ parameters and the standard postural stability test (PIGD) was observed. This result can be explained because of the different physical quantity and derived parameters considered by the two approaches: CoM displacements in our case, derivative of CoM accelerations in [41]. On the other hand, the CoM parameters in [40] have a closer physical relationship with ours: respect to us, the authors did not find a significant correlation between the PIGD scores and the parameters they selected, but this could probably be due to the exclusion of the retropulsion task from their analysis. In conclusion, we found that the PS_COM_ parameters are related to PIGD score and are also statistically significant: in fact, they clearly discriminate PD subjects from healthy controls, supporting the initial hypothesis of a worsening of PD stability during the execution of secondary tasks.

The automated assessment of UPDRS tasks is performed by means of kNN, MLR and SVM supervised classifiers, trained on the sets of selected parameters and the corresponding UPDRS scores from reference datasets of performances of PD and HC cohorts. In general, the accuracy of the SVM classifiers is better than those of the MLR and kNN classifiers. Besides, the binary-classification (i.e., HC versus PD) gives quite better results than the multiclass-classification, as expected. Moreover, in the last case, the classification error for the optimized SVM was never greater than 1 UPDRS class for all the tasks, and on the average well below of this value. This indicates that chosen classifiers are robust and, in any case, they do not make assessments too far from neurologists. Furthermore, these results agree with Table 10 about the measure of the inter-rater agreement ICC_N12-SY_, which indicate that the system performs almost as a third neurologist, except for PS_PIGD_ task. For this task, the lower value of ICC_N12-SY_ as compared to ICC_N12_ can be due to CoM parameters that are not directly comparable to PIGD subscale assessments or to the limited number of PD subjects included in the training set. 

Due to the novelty of our approach, based on low-cost optical RGB-D device, we cannot compare directly the results of the classification accuracy with other similar works. Furthermore, a limited attention has been devoted to the automated assessment of specific UPDRS tasks by motion capture technologies. Then, we decided to refer to approaches based on wearable devices employing supervised classifiers [63]. Even if not directly comparable with our tasks, Timed Up and Go (TUG) test in [64] discriminate PD from HC by machine learning approach, with accuracy of 77.5%, which is lower than the value we have obtained (Table 11). In [6] the accuracy values for the multiclass classification of the LA and AC tasks are about of 43%, which are lower than ours (Table 11), even if care must be taken because the number of classes considered is different.

Summarizing, to our knowledge this is the first time that posture instability and lower limb motor tasks were assessed with reference to the clinical UPDRS context by a system based on optical RGB-D device. The results on the classifier accuracies and on ICC show that the automated assessments of the system are comparable with the clinical ones, then demonstrating their effectiveness. Furthermore, it is also the first time that a system based on low-cost optical device characterizes CoM movements for the assessment of Parkinson’s Disease. Finally, another original feature is the interpretation of the posture improvement during quite stance as secondary motor task, and the findings about its effectiveness in assessing postural instability in PD subjects. 

Certainly, some aspects of this work require a further investigation. For instance, the number of analyzed subjects should be increased to obtain a more robust characterization of each single task and a better accuracy in the automated assessments. Furthermore, the PD subjects should be distinguished in phenotypes to verify if different sets of parameters could characterize different subtypes of parkinsonians; other balance tests should be considered to assess balance instability. These will be the next steps of our activity; the current findings encourage us to continue along this line of research to achieve a comprehensive system for the automatic and reliable assessment of PD status, suitable for the home monitoring of disease progression. 

### Limitations

Recently Microsoft announced that the Kinect device was discontinued [65], even if there is a cooperation with Intel to provide a transition from Kinect to Intel RealSense [66] or Orbbec cameras [67]. Even though our current implementation relies on the Kinect for body tracking, the Orbbec SDK or the sensor independent NUI Tracker middleware [68] are equivalent replacements for the purpose of this work. Furthermore, according to the specifications, Intel RealSense D415 combined with the NUI Tracker environment can output skeleton information at a double rate (60 fps) respect to Kinect device, providing more accuracy for fast movements.

## 7. Conclusions

In this paper, a self-managed system for the automated assessment of Parkinson’s disease at home is presented. The automated assessment is focused on lower limbs, posture and postural stability tasks as specified by standard clinical assessment scales. A high usability of the system is guaranteed to motor impaired users by a gesture based human computer interface. The patient movements are characterized by sets of selected kinematic parameters which best correlate with clinical UPDRS scores, collected in an experimental campaign conducted on PD subjects. The data acquired have been used to train supervised classifiers employed for the automated assessment of new task instances. For the first time, in the context of Parkinson’s disease, low-cost optical tracking devices are used to characterize center of mass movements as an index of postural instability. Preliminary results on the assessment accuracy, as compared to standard clinical evaluations, suggest that the proposed system is suitable for an objective assessment of posture and lower limb UPDRS tasks, also in a domestic environment, and then it could be the basis for the development of neuromonitoring and neurorehabilitation applications in a telemedicine framework.

## Figures and Tables

**Table 1:** Markers of the optoelectronic system for the accuracy estimation.

Markers	Definitions	Positions Reference
C7	7th Cervical Vertebrae	Spinous process of the 7th cervical vertebrae
LPSI/RPSI	Left/Right PSIS	Placed over Left/Right posterior superior iliac spine
LSHO/RSHO	Left/Right Shoulder	Placed on Left/Right acromioclavicular joint
LASI/RASI	Left/Right ASIS	Placed over Left/Right anterior superior iliac spine
LKNE/RKNE	Left/Right Knee	Placed on lateral epicondyle of the Left/Right knee
LANK/RANK	Left/Right Ankle	Placed on lateral malleolus along an imaginary line that passes through the trans-malleolar axis
M_HEAD_	Head	Placed on head (additional marker)
ML_WRS_/MR_WRS_	Left/Right Wrist	Placed on Left/Right wrist (additional markers)

**Table 2:** Correspondences between body segments for Kinect and optoelectronic systems.

Parameter	Kinect Segments	Optoelectronic Segments
ANG_KNEE_ Left/Right	HipL/HipR-KneeL/R	LASI/RASI-LKNE/RKNE
	AnkleL/R-KneeL/R	LANK/RANK-LKNE/RKNE
ANG_TRUNK_	SpineS-SpineB	C7-MeanPSI ^a^
ANG_FORHEAD_	Head-SpineS	M_HEAD_-C7
ANG_LATHEAD_	Head-SpineS	M_HEAD-_C7
CoM	Head-SpineS ShouldR-WristR ShouldL-WristL SpineS-SpineB HipR-AnkleR HipL-AnkleL	M_HEAD_-C7 RSHO-MR_WRS_ LSHO-ML_WRS_ C7-MeanPSI ^a^ RASI-RANK LASI-LANK

^a^ MeanPSI = (LPSI + RPSI)/2.

**Table 3:** Distribution of the severity scores among the UPDRS tasks.

	UPDRS Severity Scores		
UPDRS Task	UPDRS1 (Slight)	UPDRS2 (Mild)	UPDRS3 (Moderate)
LA^a^	16	22	18
AC	12	11	5
Gait	12	8	8
PS_retrop_	8	6	14
Po	14	8	6

^a^ In the LA task, both legs were assessed.

**Table 4:** Mean and standard deviation of the Pearson’s correlation coefficients for essential parameters estimated by the two systems.

Parameter	Pearson’s Correlation Coefficient *r* ^a^	
	Mean ± Std. Dev.	*p*-Value^a^
ANG_KNEE_	0.94 ± 0.07	9.09 × 10^−3^
ANG_TRUNK_	0.87 ± 0.10	6.72 × 10^−3^
ANG_FORHEAD_	0.73 ± 0.20	3.98e × 10^−2^
ANG_LATHEAD_	0.71 ± 0.23	3.57 × 10^−2^
CoM_AP_	0.84 ± 0.11	3.18 × 10^−3^
CoM_ML_	0.90 ± 0.09	8.94 × 10^−3^

^a^ Significance level *p* < 0.05.

**Table 5:** Parameters of the LA task: discriminant power and correlation with UPDRS scores.

		Mann-Whitney U Test				Spearman Coefficient	
Name	Meaning (Unit)	Median HC	Median PD	Z	*p*-Value ^a^	ρ	*p*-Value ^a^
MKAm	Mean of Maximum Knee Angle (degree)	32.41	25.02	1.93	5.37 × 10^−2^	−0.72	9.99 × 10^−6^
MKAv	Var. of Maximum Knee Angle (-)	0.07	0.13	1.81	7.03 × 10^−2^	0.49	6.72 × 10^−3^
TDm	Mean of movement Total Duration (s)	0.26	0.42	2.88	3.95 × 10^−3^	0.43	1.98 × 10^−2^
TDv	Var. of movement Total Duration (-)	0.10	0.12	1.68	9.19 × 10^−2^	0.43	2.07 × 10^−2^
SPm	Mean Speed of movement (degree/s)	114.8	64.20	3.00	2.66 × 10^−3^	−0.84	8.18 × 10^−9^
PM	Num. of poor movements (#)	0.00	1.00	1.99	4.69 × 10^−2^	0.74	3.94 × 10^−6^

^a^ Significance level *p* < 0.05.

**Table 6:** Parameters of the AC task: discriminant power and correlation with UPDRS scores.

		Mann-Whitney U Test				Spearman Coefficient	
Name	Meaning (Unit)	Median HC	Median PD	Z	*p*-Value ^a^	ρ	*p*-Value ^a^
MBA	Maximum Bending Angle (degree)	17.50	31.26	3.18	1.44 × 10^−3^	0.75	4.00 × 10^−7^
TD	Total Duration of S2S movement (s)	0.90	2.42	2.86	4.17 × 10^−3^	0.80	1.08 × 10^−8^
SPm	Mean Speed of S2S movement (degree/s)	21.85	12.92	2.76	5.84 × 10^−3^	-0.69	6.26 × 10^−6^
NPeaks	Number of Bending Peaks (#)	1.00	1.00	1.13	2.59 × 10^−1^	0.63	5.65 × 10^−5^

^a^ Significance level *p* < 0.05.

**Table 7:** Parameters of the Po task: discriminant power and correlation with UPDRS scores.

		Mann-Whitney U Test				Spearman Coefficient	
Name	Meaning (Unit)	Median HC	Median PD	Z	*p*-Value ^a^	ρ	*p*-Value ^a^
FTB	Forward Trunk Bending (degree)	0.38	−5.69	2.71	9.88 × 10^−4^	−0.70	1.36 × 10^−4^
FTB_Δ_	Var. of Forward Trunk Bending (degree)	0.35	0.27	0.18	8.55 × 10^−1^	0.43	5.54 × 10^−2^
FHB	Forward Head Bending (degree)	−1.83	−6.86	1.92	5.23 × 10^−2^	−0.78	5.90 × 10^−6^
FHB_Δ_	Var. of Forward Head Bending (degree)	0.46	0.53	0.22	8.17 × 10^−1^	0.27	3.62 × 10^−1^
LHB	Absolute Lateral Head Bending (degree)	2.05	3.02	0.53	6.07 × 10^−1^	0.59	2.39 × 10^−3^
LHB_Δ_	Var. of Lateral Head Bending (degree)	0.19	0.43	1.53	1.25 × 10^−1^	0.43	6.54 × 10^−2^

^a^ Significance level *p* < 0.05.

**Table 8:** Parameters of the PS_COM_ task: discriminant power and correlation with UPDRS scores.

		Mann-Whitney U Test				Spearman Coefficient ^b^	
Name	Meaning (Unit)	Median HC	Median PD	Z	*p*-Value ^a^	ρ	*p*-Value ^a^
APr	CoM AP sway Range (cm)	0.59	1.13	1.80	7.20 × 10^−2^	0.59	3.24 × 10^−3^
APt	CoM AP sway Total (cm)	1.49	3.28	2.23	2.50 × 10^−2^	0.65	2.54 × 10^−2^
MLt	CoM ML sway Total (cm)	0.98	3.48	2.24	2.53 × 10^−2^	0.48	1.88 × 10^−2^
APv	CoM AP sway Velocity (cm/s)	0.72	1.32	1.86	6.34 × 10^−2^	0.56	4.92 × 10^−2^
MLv	CoM ML sway Velocity (cm/s)	0.48	1.49	2.24	2.53 × 10^−2^	0.42	4.25 × 10^−2^
SwayArea	CoM Sway Area (cm^2^)	0.30	0.85	1.58	1.13 × 10^−1^	0.59	2.92 × 10^−3^

^a^ Significance level *p* < 0.05; ^b^ The Spearman correlation was evaluated respect to the PS_PIGD_ subscale scores.

**Table 9:** Average differences of CoM parameters between Phase1 and Phase2 of the Po task for HC and PD subjects.

	PD Subjects	HC Subjects	Mann-Whitney U Test		*T* Test
Name	Phase2-Phase1	Phase2-Phase1	Z	*p*-Value ^a^	*p*-Value ^a^
APt	1.61	0.54	2.04	4.17 × 10^−2^	2.22 × 10^−3^
MLt	1.87	0.27	2.04	4.17 × 10^−2^	6.33 × 10^−3^
APv	1.14	0.52	2.11	3.44 × 10^−2^	9.86 × 10^−4^
MLv	1.04	0.45	1.97	4.89 × 10^−2^	1.11 × 10^−3^
SwayArea	1.88	0.25	2.13	3.28 × 10^−2^	7.19 × 10^−3^

^a^ Significance level *p* < 0.05.

**Table 10:** Intra Class Correlations for the system and the neurologists assessment reliability.

Reliability/ Task	LA	AC	Po	PS_PIGD_
ICC_N12_^a^	0.80	0.82	0.77	0.73
ICC_N12-SY_^a^	0.77	0.80	0.74	0.65

^a^ Significance level: *p* < 0.05.

**Table 11:** Classification accuracies for the supervised classifiers.

		LEAVE-ONE-OUT		K-FOLD (10) ^a^	
Task	Classifier	HC-PD (2-Classes)	UPDRS (3-Classes)	HC-PD (2-Classes)	UPDRS (3-Classes)
**LA**	SVM	95.6	68.9	96.5	73.6
	KNN (k = 3)	94.5	51.7	96.5	58.0
	MLR	89.6	68.9	89.6	70.5
**AC**	SVM	88.2	66.3	88.2	69.9
	KNN (k = 3)	86.0	60.0	88.2	67.5
	MLR	94.1	70.5	96.8	73.3
**Po**	SVM	91.6	68.0	93.5	68.2
	KNN (k = 3)	95.8	70.8	95.0	68.9
	MLR	83.3	62.5	81.7	58.8
**PS_COM_**	SVM	95.2	58.3	93.2	59.6
	KNN (k = 3)	92.8	41.6	95.7	45.8
	MLR	95.8	50.0	91.9	52.1

^a^ 100 iterations.
